# Derivatives of Imidazole and Carbazole as Bifunctional Materials for Organic Light-Emitting Diodes

**DOI:** 10.3390/ma15238495

**Published:** 2022-11-29

**Authors:** Oleksandr Bezvikonnyi, Ronit Sebastine Bernard, Viktorija Andruleviciene, Dmytro Volyniuk, Rasa Keruckiene, Kamile Vaiciulaityte, Linas Labanauskas, Juozas Vidas Grazulevicius

**Affiliations:** 1 Department of Polymer Chemistry and Technology, Faculty of Chemical Technology, Kaunas University of Technology, K. Baršausko g. 59, LT-51423 Kaunas, Lithuania; 2 Department of Physics, Faculty of Mathematics and Natural Science, Kaunas University of Technology, Studentų g. 50, LT-51369 Kaunas, Lithuania; 3 Center for Physical Sciences and Technology (FTMC), Department of Organic Chemistry, Sauletekio Ave. 3, LT-10257 Vilnius, Lithuania

**Keywords:** imidazole, carbazole, luminescence, emitter, host, organic light-emitting diode, upconversion

## Abstract

New derivatives of carbazole and diphenyl imidazole for potential multiple applications were synthesized and investigated. Their properties were studied by thermal, optical, photophysical, electrochemical, and photoelectrical measurements. The compounds exhibited relatively narrow blue light-emission bands, which is favorable for deep-blue electroluminescent devices. The synthesized derivatives of imidazole and carbazole were tested as fluorescent emitters for OLEDs. The device showed deep-blue emissions with CIE color coordinates of (0.16, 0.08) and maximum quantum efficiency of 1.1%. The compounds demonstrated high triplet energy values above 3.0 eV and hole drift mobility exceeding 10^−4^ cm^2^/V·s at high electric fields. One of the compounds having two diphenyl imidazole moieties and *tert*-butyl-substituted carbazolyl groups showed bipolar charge transport with electron drift mobility reaching 10^−4^ cm^2^/V·s at electric field of 8 × 10^5^ V/cm. The synthesized compounds were investigated as hosts for green, red and sky-blue phosphorescent OLEDs. The green-, red- and sky-blue-emitting devices demonstrated maximum quantum efficiencies of 8.3%, 6.4% and 7.6%, respectively.

## 1. Introduction

Organic light-emitting diodes (OLEDs) are used in displays and lighting devices due to their low turn-on voltage, high power efficiency, mechanical flexibility and wide range of colors [1]. Generally, electrons and holes injected from the respective electrodes move through charge injection/transport layers into the emitting layer of a device. There, electrons and holes recombine and form singlet and triplet excitons [2]. Fluorescent OLEDs exploit only singlet excitons for light emission [3]. Organic fluorescent materials with high emission quantum yield, restricted intermolecular interactions, and appropriate charge injection/transporting properties are required for stable and efficient OLEDs [4,5]. Because of the high stability of blue singlet emission-based OLEDs, they are still used in commercial displays [6]. Therefore, development of stable, efficient and cost-effective fluorescent blue emitters remains an urgent task. Phosphorescent OLEDs (PHOLEDs) can utilize both singlet and triplet excitons for light emission. This property allows us to increase external quantum efficiency of PHOLEDs [7,8]. Phosphorescent emitters normally have to be doped in a suitable charge transporting host matrix due to their typically poor charge carrier mobility [9]. Host materials with appropriate energy levels and triplet energies, balanced charge carrier transport, as well as high thermal stability and morphological stability of their layers, can enable excellent PHOLED performance [10,11,12].

Many low-molar-mass electroactive compounds were synthesized for highly efficient OLEDs [13]. The advantages of low-molar-mass compounds are their precise chemical structure, and high purity [14]. Among the wide-bandgap materials, carbazole derivatives are among the most widely studied due to their good hole-transporting properties, high triplet energy and rigid molecular framework [15]. Carbazole ring can be easily modified by attaching various electron-accepting units, such as imidazole [16]. Derivatives of carbazole with benzimidazole [12,17] or -phenanthro [9,10-d]imidazole [18] substituents demonstrate suitable triplet energies, efficient bipolar charge transport and relatively high photoluminescence quantum efficiencies. Blue OLEDs, based on such emitters, demonstrate external quantum efficiency, reaching 3% [17,18]. Blue emitters can sometimes be utilized also as hosts to generate green or white light by energy transfer to an emissive compound [19,20,21]. Donor–acceptor derivatives of imidazole containing diphenylimidazole [22,23], pyrene-imidazole [24,25,26], phenanthroimidazole [26,27,28,29,30,31,32,33,34], benzimidazole [35,36], and theobromine [37] exhibit specific localization of molecular orbitals due to highly twisted molecular geometry and conformations. Consequently, these compounds can form a hybridized local charge transfer state (HLCT) [22,23,24,25,26,27,28,29,30,31,32,33,34,35,36,37] that can be exploited for practical electroluminescent applications, including blue-emitting OLEDs. Recently, there has been a great deal of interest in the use of HLCT compounds including imidazole derivatives [34,38] as hosts in systems utilizing prompt fluorescent [34], TADF [39], phosphorescent [38,39] and HLCT [40] emitters. Such an approach results in triplet harvesting via host similarly to the principle of hyperfluorescence involving TADF host matrices [41,42].

The simple and efficient synthesis of materials intended for OLEDs to a great extent contributes to the cost effectiveness of the devices [43]. Carbazole and imidazole derivatives correspond to the criterion of cost effectiveness. Carbazole derivatives are susceptible to various synthetic methods, including nucleophilic substitution, Suzuki, and Buchwald–Hartwig coupling [44]. Imidazole derivatives can be easily obtained by one-pot cyclization reaction from readily available starting compounds [45,46].

In this work, we present multifunctional derivatives of carbazole and imidazole obtained by simple one-pot condensation reaction. The appropriate properties of the obtained compounds enable them to be used as emitters for doping-free blue OLEDs and as hosts for green, red and sky-blue PHOLEDs, avoiding the photochemical oxidation of the imidazole ring by the device fabrication in an inert atmosphere.

## 2. Materials and Methods

The 9*H*-carbazole (95%, Reachem Slovakia, Bratislava, Slovakia), benzil (98%, Aldrich, St. Louis, MO, USA), 9-ethyl-9*H*-carbazole-3 carbaldehyde (98%, Aldrich), 9-ethyl-9*H*-carbazol-3-amine (98%, Aldrich), ammonium acetate (98%, Fluka, Dresden, Germany), acetic acid (99%, Reachem), anhydrous sodium sulfate (99%, Reachem), *tert*-butyl chloride (99%, Aldrich), aluminum(III) chloride (99%, Aldrich), 2-ethylhexyl bromide (95%, Aldrich), and tin(II) chloride (98%, Aldrich) were purchased as reagent grade chemicals and used as received.

3,3′-(4,5-Diphenyl-1*H*-imidazole-1,2-diyl)bis(9-ethyl-9*H*-carbazole) (**1**)

A mixture of benzil (1 g, 4.8 mmol), 9-ethyl-9*H*-carbazole-3-carbaldehyde (1.1 g, 4.8 mmol), 9-ethyl-9*H*-carbazol-3-amine (1.2 g, 5.7 mmol) and ammonium acetate (3.7 g, 47.6 mmol) in acetic acid (5 mL) was heated at the reflux temperature for 1 h. The mixture was poured into cold water and extracted with chloroform. The organic layer was dried with anhydrous sodium sulfate, filtered and distilled. The product was purified by column chromatography using a mixture of acetone and hexane at a volume ratio of 1:3 as an eluent. The compound **1** was crystallized from acetone. The yield of pale-yellow crystals was 2.38 g (82%), mp = 264–265 °C.

IR, cm^−1^: 3055 (ar. C-H), 2978 (aliph. C-H), 1599, 1470, 1444 (ar. C=C), 1332, 1230 (C-N).

^1^H NMR (400 MHz, CDCl_3_-d, δ): 8.28 (d, J = 1.3 Hz, 1H, ar.), 7.84 (d, J = 7.8 Hz, 1H, ar.), 7.76–7.78 (m, 2H, ar.), 7.62 (d, J = 7.3 Hz, 2H, ar.), 7.37–7.44 (m, 2H, ar.), 7.30–7.33 (m 2H, ar.), 7.20–7.26 (m, 4H, ar.), 7.12–7.18 (m, 5H, ar.), 7.02–7.10 (m, 5H, ar.), 4.25 (q, J = 7.2 Hz, 2H, CH_2_), 4.17 (q, J = 7.2 Hz, 2H, CH_2_), 1.34 (t, J = 7.2 Hz, 3H, CH_3_), 1.26 (t, J = 7.2 Hz, 3H, CH_3_).

^13^C NMR (101 MHz, CDCl_3_-d, δ): 140.54, 140.26, 139.13, 131.24, 131.18, 128.29, 128.21, 127.74, 126.82, 126.36, 126.11, 125.74, 123.07, 122.70, 122.49, 120.81, 120.67, 120.60, 119.23, 119.03, 108.82, 108.55, 108.49, 107.95, 37.78, 37.58, 30.89, 13.79.

MS (APCl^+^), *m/z* = 608 [M + H]^+^.

6,6′-(4,5-Diphenyl-1*H*-imidazole-1,2-diyl)bis(3-(*tert*-butyl)-9-ethyl-9*H*-carbazole) (**2**)

A solution of compound **1** (1 g, 1.7 mmol) and aluminum(III) chloride (0.8 g, 6.6 mmol) in dichloromethane (10 mL) was cooled down using an ice bath. The solution of *tert*-butyl chloride (0.4 mL, 3.6 mmol) in dichloromethane (5 mL) was added dropwise to the reaction mixture. The mixture was stirred at room temperature for 1 h, then poured into water and extracted with chloroform. The organic layer was dried with anhydrous sodium sulfate, filtered and distilled. The product was purified by column chromatography using a mixture of acetone and hexane at a volume ratio of 1:6 as an eluent. The yield of pale-yellow solid was 0.82 g (69%).

IR, cm^−1^: 3053 (ar. C-H), 2963 (aliph. C-H), 1604, 1481 (ar. C=C), 1301, 1232 (C-N).

^1^H NMR (400 MHz, DMSO-d6, δ): 8.20–8.34 (m, 2H, ar.), 8.10–8.13 (m, 1H, ar.), 7.94–8.05 (m, 1H, ar.), 7.68–7.76 (m, 1H, ar.), 7.57–7.60 (m, 2H, ar.), 7.50–7.56 (m, 3H, ar.), 7.45–7.49 (m 2H, ar.), 7.33–7.42 (m, 4H, ar.), 7.16–7.31 (m, 6H, ar.), 4.25–4.43 (m, 4H, CH_2_), 1.18–1.38 (m, 24H, CH_3_).

^13^C NMR (101 MHz, DMSO-d6, δ): 149.73, 148.06, 142.30, 141.90, 140.55, 139.59, 138.90, 138.74, 138.51, 136.92, 136.83, 135.43, 132.06, 132.03, 131.64, 131.48, 128.84, 128.59, 126.96, 126.70, 126.31, 125.89, 124.90, 124.83, 124.13, 123.04, 122.95, 122.17, 122.13, 121.88, 121.85, 121.80, 120.63, 120.19, 119.96, 119.79, 117.33, 116.04, 109.58, 109.43, 109.33, 109.03, 108.88, 106.02, 37.64, 37.48, 37.21, 35.48, 35.42, 34.86, 34.53, 32.17, 32.02, 14.27, 14.20, 14.15.

MS (APCl^+^), *m/z* = 720 [M + H]^+^.

3,3′-(1,1′-(9-(2-Ethylhexyl)-9*H*-carbazole-3,6-diyl)bis(4,5-diphenyl-1*H*-imidazole-2,1-diyl))bis(9-ethyl-9*H*-carbazole) (**3**)

Compound **3** was synthesized from benzil (1.35 g, 6.4 mmol), 9-ethyl-9*H*-carbazole-3-carbaldehyde (1.59 g, 7.1 mmol), diaminocarbazole 2 (1 g, 3.2 mmol) and ammonium acetate (4.98 g, 64.6 mmol) by the same procedure as compound **1**. The product was purified by column chromatography using the mixture of acetone and hexane at a volume ratio of 1:4 as an eluent. The yield of white solid was 2.63 g (74%).

IR, cm^−1^: 3054 (ar. C-H), 2932 (aliph. C-H), 1602, 1475 (ar. C=C), 1333, 1231 (C-N).

^1^H NMR (400 MHz, CDCl_3_-d, δ): 8.16 (s, 2H, ar.), 7.69 (d, J = 7.7 Hz, 2H, ar.), 7.60–7.50 (m, 6H, ar.), 7.43 (d, J = 8.5 Hz, 2H, ar.), 7.33 (t, J = 7.6 Hz, 2H, ar.), 7.26 (d, J = 8.2 Hz, 2H, ar.), 7.18 (t, J = 7.5 Hz, 6H, ar.), 7.14–7.07 (m, 7H, ar.), 7.07–7.00 (m, 7H, ar.), 6.89–7.00 (m, 4H, ar.), 4.10–4.24 (m, 4H, CH_2_), 3.94–4.02 (m, 2H, CH_2_), 1.84–1.91 (m, 1H, CH), 1.35–0.98 (m, 14H, CH_2_, CH_3_), 0.73 (t, J = 7.4 Hz, 3H, CH_3_), 0.68 (t, J = 6.9 Hz, 3H, CH_3_).

^13^C NMR (101 MHz, CDCl_3_-d, δ): 148.33, 140.50, 140.24, 139.62, 137.94, 134.87, 131.25, 131.16, 131.05, 129.32, 128.14, 127.50, 126.88, 126.77, 126.41, 125.73, 123.06, 122.64, 122.31, 121.63, 121.44, 120.66, 120.44, 119.01, 109.46, 108.51, 107.87, 57.27, 47.85, 39.16, 37.69, 30.91, 28.51, 24.37, 22.87, 13.90, 10.89.

MS (APCl^+^), *m/z* = 1103 [M + H]^+^.

6,6′-(1,1′-(9-(2-Ethylhexyl)-9*H*-carbazole-3,6-diyl)bis(4,5-diphenyl-1*H*-imidazole-2,1-diyl))bis(3-(*tert*-butyl)-9-ethyl-9*H*-carbazole) (**4**)

Compound **4** was synthesized from compound **3** (1 g, 0.9 mmol), AlCl_3_ (0.48 g, 3.6 mmol) and *tert*-butyl chloride (0.22 mL, 2 mmol) by the same procedure as compound **2**. The product was purified by column chromatography using the mixture of acetone and hexane at a volume ratio of 1:5 as an eluent. The yield of pale-yellow solid was 0.62 g (57%).

IR, cm^−1^: 3052 (ar. C-H), 2958 (aliph. C-H), 1605, 1480 (ar. C=C), 1300, 1234 (C-N).

^1^H NMR (400 MHz, CDCl_3_-d, δ): 8.36 (s, 2H, ar.), 7.85 (s, 2H, ar.), 7.56 (d, J = 7.6 Hz, 4H, ar.), 7.50 (s, 2H, ar.), 7.42 (d, J = 8.5 Hz, 2H, ar.), 7.23–7.15 (m, 8H), 7.07–7.15 (m, 8H, ar.), 7.02–7.07 (m, 4H, ar.), 6.89–7.01 (m, 6H, ar.), 4.07–4.17 (m, 4H, CH2), 3.95–4.02 (m, 2H, CH_2_), 1.83–1.92 (m, 1H, CH), 1.30 (s, 18H, CH_3_), 1.27–1.11 (m, 11H, CH_2_, CH_3_), 0.99–1.09 (m, 3H, CH_3_), 0.77 (t, J = 7.4 Hz, 3H, CH_3_), 0.67 (t, J = 6.5 Hz, 3H, CH_3_).

^13^C NMR (101 MHz, CDCl_3_-d, δ): 148.53, 146.00, 142.10, 140.54, 139.93, 138.43, 138.01, 134.94, 133.67, 131.24, 131.14, 130.85, 129.39, 129.36, 128.14, 128.09, 127.50, 127.48, 126.40, 126.27, 123.98, 123.66, 123.17, 123.04, 122.99, 122.75, 122.68, 122.40, 121.68, 120.64, 116.79, 109.30, 107.99, 107.39, 38.97, 37.67, 34.38, 31.95, 24.58, 22.64, 13.90, 10.91.

MS (APCl^+^), *m/z* = 1216 [M + H]^+^.

## 3. Results and Discussions

### 3.1. Synthesis

**Scheme 1:**
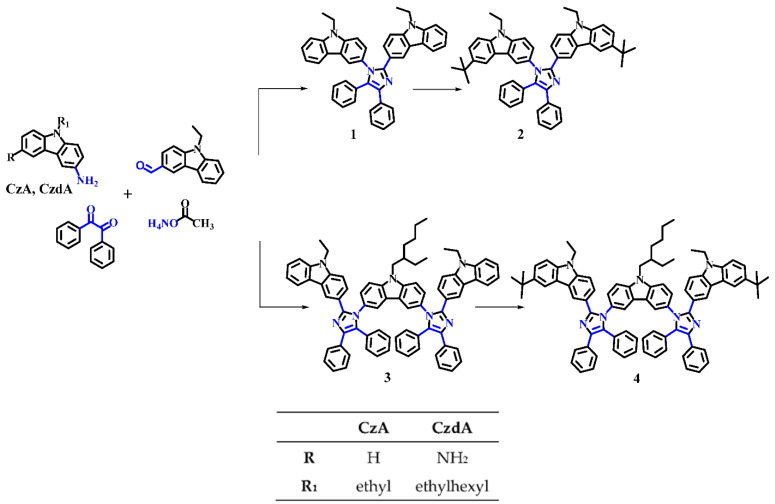
Synthesis of derivatives of carbazole and diphenyl imidazole.

The synthetic routes for preparation of derivatives of carbazole and diphenyl imidazole are presented in Scheme 1. The target compound **1** was synthesized using the commercial reagents. During the condensation reaction, a *tetra*-substituted imidazole ring was obtained. *Tert*-butyl groups were attached to carbazole moieties to obtain compound **2**. Compounds **3** and **4** were prepared by the same methods as **1** and **2**, respectively.

The yields of derivatives **1** and **3** were of 82% and 74%, respectively, whereas the Friedel–Crafts alkylations gave compounds **2** and **4** in 69% and 57% yields, respectively. All the compounds were found to be soluble in common organic solvents. Their structures were confirmed by ^1^H NMR, ATR-IR, and mass spectrometry.

### 3.2. Thermal Properties

The behavior of compounds **1**–**4** under heating was studied by TGA and DSC. The obtained results are summarized in Table 1.

**Figure 1:**
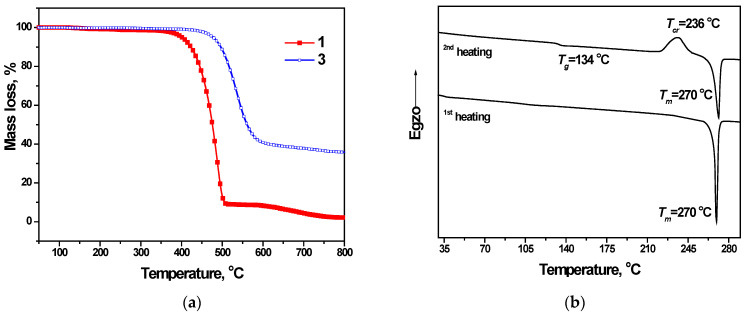
(**a**) TGA curves of compounds **1** and **3**; (**b**) DSC curves of compound **1**.

The synthesized compounds showed high thermal stability confirmed by TGA. The 5% weight-loss temperatures (*T_D_*) of the compounds with one imidazole ring (**1** and **2**) were found to be ca. 400 °C, while compounds with two imidazole units (**3** and **4**) showed TD higher than 477 °C (Figure 1a and Figure S1a). Compound **1** was obtained as a crystalline substance. In the first DSC heating scan of **1** (Figure 1b), an endothermic melting signal was observed at 270 °C. The second heating scan revealed glass transition temperature (*T_g_*) at 134 °C and further heating showed crystallization and melting signals. Compounds **2**, **3** and **4** were isolated after the synthesis and purification as amorphous substances with *T_g_* of 146 °C, 151 °C and 172 °C, respectively, observed in the heating and cooling scans (Figure S1b–d). DSC results demonstrate that increased molecular weight by attachment additional carbazole–imidazole moiety or *tert*-butyl groups results in an increase of *T_g_*.

### 3.3. Photophysical Properties

**Figure 2:**
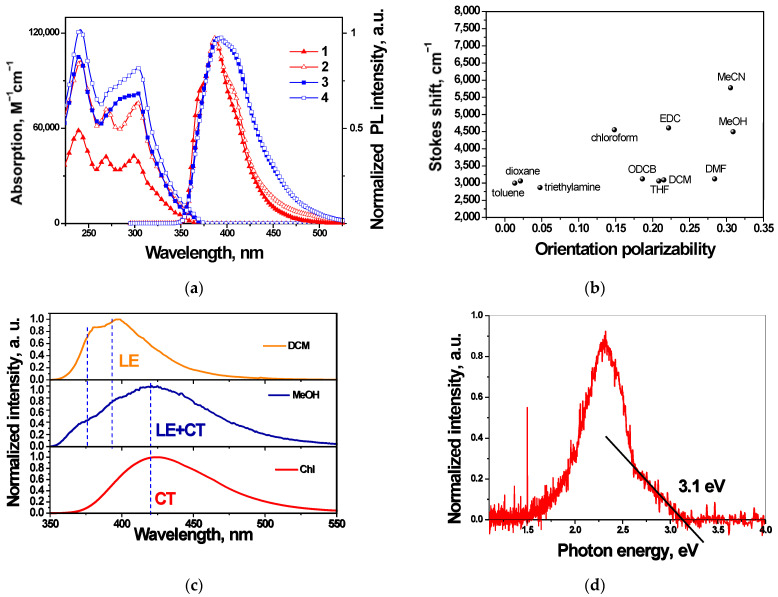
(**a**) Absorption and PL spectra of the dilute THF solutions of **1**–**4**; (**b**) the Lippert–Mataga plots for 2; (**c**) PL spectra of the dilute DCM, MeOH and chloroform solutions of **2**; (**d**) phosphorescence spectrum of the dilute THF solution of compound **4** recorded at 77 K.

Absorption spectra of dilute solutions of compounds **1**–**4** are presented in Figure 2a and Figures S2a–S5a. Optical characteristics of the compounds are collected in Table 1. The dilute THF solutions of **1**–**4** absorb UV radiation up to 380 nm. The attachment of additional fragments of carbazole and diphenyl imidazole did not contribute to the extension of the systems of conjugated π-electrons (cf. UV spectra of THF solutions of **1** and **2** with those of the solutions of **3** and **4**). The investigation of absorption and emission spectra of **1**–**4** dissolved in solvents of different polarity was carried out (Figures S2–S5). No dependence of the wavelengths of absorption peaks on the polarity of solvents was observed (Figures S2a–S5a). THF solutions of **1**–**4** emitted blue light with the photoluminescence (PL) intensity maxima in the range of 398–403 nm (Figure 2a, Table 1).

The aspecific electrostatic interactions between molecules of the solvent and the materials in the context of Onsager interpretation [47] is in the core of the Lippert–Mataga approach [48,49,50] used for the description of the solvatochromic effect. The Lippert–Mataga plots were built for solutions of **1**–**4**, which demonstrated the correlation of the Stokes shift and orientation polarizability (Figure 2b and Figure S6a–c). It was established that the Lippert–Mataga plots cannot be sufficiently fitted linearly for the solutions of **1**–**4** with appropriate value of errors. For example, the toluene and THF solutions of **1**–**4** exhibited local excited (LE) state emission solely despite of the different polarity of the solvents represented by the different values of orientation polarizability. Additionally, the bathochromic shift of charge transfer (CT) bands of **1**–**4** appeared with the increase in polarity of the solvents without a strict order. These observations point to the fact that the Lippert–Mataga approach is not applicable for the description of compounds **1**–**4** in terms of dipole moment change [51]. The interaction of the solvent and the solute is ongoing in an individual manner for the particular solvent, resulting in the different distribution of LE and CT components. Apparently, the emission of **1**–**4** in solutions is affected by HLCT, which is characterized as combined transition of the LE and CT states. The observed LE, CT and mixed LE and CT states of the solutions of compounds **1**–**4** in different solvents may originate from the twisting of the angles between the carbazole and imidazole moieties (Figure 2c) [45].

The wavelengths of intensity maxima of phosphorescence spectra of THF solutions of the compounds recorded at 77 K ranged from 533 nm to 537 nm (Figure 2d and Figure S7a–c). The triplet energies (*E_T_*) of the compounds were estimated from the onset of the phosphorescence spectra (Figure 2d). Compound **1** exhibited the highest *E_T_* of 3.18 eV, while compounds **2**, **3** and **4** exhibited slightly smaller triplet energy levels 3.16 eV, 3.08 eV and 3.10 eV, respectively (Table 1).

**Figure 3:**
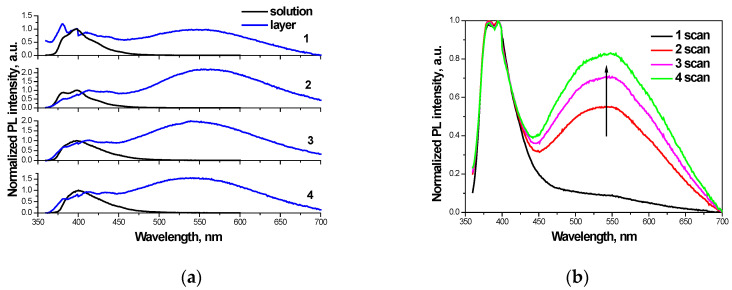
(**a**) PL of the dilute THF solutions and the layers of **1**–**4**; (**b**) PL spectra of the solid solution of **1** in ZEONEX 480 after UV-irradiation for 10 min after every measurement.

The emission spectra of the layers of compounds **1**–**4** had two peaks at ca. 400 nm and a wide red-shifted emission peak at ~550 nm (Figure 3a). It is known that an imidazole ring in the presence of oxygen under UV-irradiation is subjected to the ring-opening reaction, which results in the formation of new materials [52,53]. The PL spectra of 1 wt.% solid solution of compound **1** in ZEONEX 480 were recorded after different periods of UV irradiation. They showed consistent increase in intensity of emission band, peaking at 550 nm (Figure 3b). The small concentration of compound **1** in ZEONEX 480 ensures absence of intermolecular interactions as well as formation of intermolecular excimers. PL decay curves of the layers of **1**–**4** recorded at the different wavelengths of ca. 410 nm and ca. 550 nm exhibited prompt fluorescence (Figure S8). The observed difference between PL lifetimes demonstrates the different origin of emission. Thus, PL decay curves recorded at 550 nm have a biexponential character. The slightly longer-lived component of the prompt fluorescence is attributed to the oxidized product. Thus, these experiments prove the photochemical process occurring in the layers of compounds **1**–**4** [54]. The photochemical process was also detected for the THF solutions of the compounds (Figure S9a–c). It was manifested by substantial decrease of intensity and the redshift of the peak after continuous UV irradiation.

### 3.4. Electrochemical and Photoelectrical Properties

The values of ionization potential (IP) estimated by cyclic voltammetry (CV) and UV photoelectron (PE) spectroscopy are presented in Table 2. The IP_CV_ values of the compounds were estimated from the half-wave potential of the first oxidation relative to ferrocene (Figure S10a–d). The electron affinity (EA_CV_) values were obtained from the IP_CV_ values and the optical bandgaps, which were deduced from the edges of the absorption spectra of the dilute THF solutions of **1**–**4**. The IP_CV_ and EA_CV_ values of **1**–**4** are in the small ranges of 5.21–5.26 eV and 1.81–1.94 eV, respectively. The comparable values of the IP_CV_ and EA_CV_ observed for all the studied compounds demonstrate the same oxidation and reduction sites.

**Figure 4:**
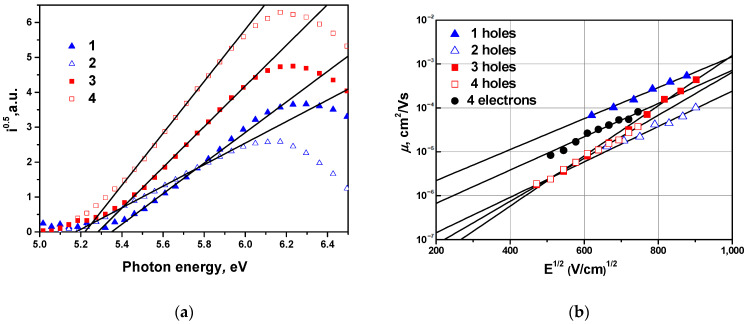
(**a**) Photoelectron emission spectra of the layers of compounds **1**–**4**; (**b**) electric field dependence of hole and electron mobilities for the layers of compounds **1**–**4**.

The photoelectron emission spectra of the solid samples of **1**–**4** are presented in Figure 4a. The IP_PE_ values of the compounds are in the slightly larger range of 5.18–5.35 eV comparing with that of IP_CV_. This observation can be explained by the different environments in solutions and solid layers of the compounds. IP_CV_ and IP_PE_ values of compounds **2** and **4**, bearing *tert*-butyl groups, are slightly lower than those of their counterparts **1** and **3**, respectively. This trend can be explained by the σ donor effect of *tert*-butyl groups [55] which reduce the ionization potential values.

The TOF technique was used to study charge transporting properties of the compounds. TOF current transients for holes and electrons for vacuum deposited films of the compounds **1**–**4** were recorded at different electric fields (Figure S11). When the transit times were well recognized from the TOF current transients in log–log scales, charge mobilities were calculated. The electric field dependence values of hole and electron mobilities of the layers of compounds **1**–**4** are shown in Figure 4b. The layers of compounds demonstrated hole drift mobilities ranging from 10^−5^ cm^2^/V·s to 10^−4^ cm^2^/V·s at electric field of 6.4 × 10^5^ V/cm (Table 2). Electron mobility was detected only in the layer of compound **4**, which reached 10^−4^ cm^2^/V·s at high electric fields (>8.1 × 10^5^ V/cm). Thus, the difference between hole and electron mobilities in the layer of compound **4** is only one order of magnitude. To achieve high efficiency of PHOLED, host materials with a balanced charge transport are required [56]. This allows generation of a broad charge recombination zone in the emissive layer.

### 3.5. Performance in Fluorescent OLEDs

**Figure 5:**
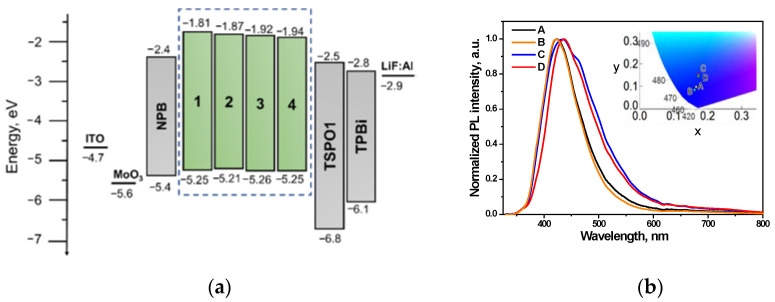

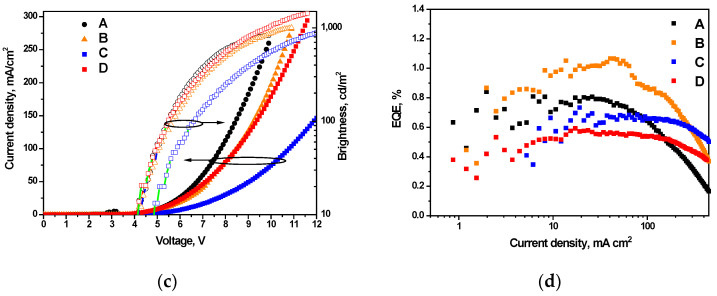
Diagram of energy levels of OLEDs A–D (**a**); electroluminescence spectra of OLEDs A–D recorded at applied voltage of 10 V; inset shows CIE 1931 color diagram with the corresponding CIE color coordinates (**b**); current density and luminance versus voltage (**c**); external quantum efficiency versus current density (**d**). Arrow denotes the correspondence of data to axis.

To investigate electroluminescent properties of compounds **1**–**4** as materials for doping-free light-emitting layers (EML), OLEDs A–D were fabricated using device structure ITO/MoO_3_ (1 nm)/NPB (55 nm)/EML (40 nm)/TSPO1 (5 nm)/TPBi (65 nm)/LiF (0.5 nm)/Al. A diagram of energy levels of devices A–D is shown in Figure 5a. The electroluminescent characteristics are summarized in Table 3.

In OLEDs, the layers of MoO_3_ and LiF were used as the charge injections layers. The layer of *N*,*N*′-di(1-naphthyl)-*N*,*N*′-diphenyl-(1,1′-biphenyl)-4,4′-diamine acted as the hole-transporting layer. The layers of diphenyl [4-(triphenylsilyl)phenyl]phosphine oxide (TSPO1) and 2,2′,2″-(1,3,5-benzinetriyl)-tris(1-phenyl-1-*H*-benzimidazole) (TPBi) were utilized as the hole/exciton blocking and electron transporting layers, respectively.

Electroluminescence (EL) spectra of OLEDS A–D are shown in Figure 5b and Figure S12. Electroluminescence intensity maxima of devices A–D were observed in the range of 418–437 nm. According to the CIE color coordinates, A, B and C, D emitted deep-blue and blue light, respectively (Figure 5b inset, Table 3). The shapes and positions of the peaks of electroluminescence spectra of the devices were very similar to those of the fluorescence spectra of the solutions of compounds **1**–**4** in THF (Figure 2a and Figure 5b). The shapes of EL spectra were practically the same at different applied voltages (Figure S12). The emission peaks of devices C and D were wider and slightly red-shifted compared to those of devices A and B. It was also observed that during operation of the devices at various electrical voltages, no additional emission peak appeared in the longer wavelength region.

All OLEDs showed relatively low turn-on voltages (V_on_) of 4.0–4.8 V (Figure 5c). This observation confirms that injection and transport of holes and electrons towards the emission layer was efficient. Device D showed maximum brightness of 1430 cd/m^2^ and minimum turn-on voltage of 4.0 V. Device B, with emissive layer of emitter **2**, demonstrated the best electroluminescence properties (Figure 5b,c and Figure S13). Maximum current efficiency (0.8 cd/A) and maximum energy efficiency (0.42 lm/W) of device B were similar to those of devices A, C and D. However, the maximum external quantum efficiency (EQE) of device B was the highest one (1.1%) (Figure 5d). This observation can be attributed to the narrowest EL spectrum of device B (Figure 5b).

The obtained results show that the synthesized derivatives of carbazole and diphenyl imidazole (**1**–**4**) are suitable for the formation of functional layers of OLED. The photo-oxidation process of the imidazole ring can be successfully suppressed by fabrication of the devices in an inert atmosphere. This is confirmed by the absence of the additional low-energy peaks in the electroluminescence spectra.

### 3.6. Performance in Phosphorescent OLEDs

**Figure 6:**
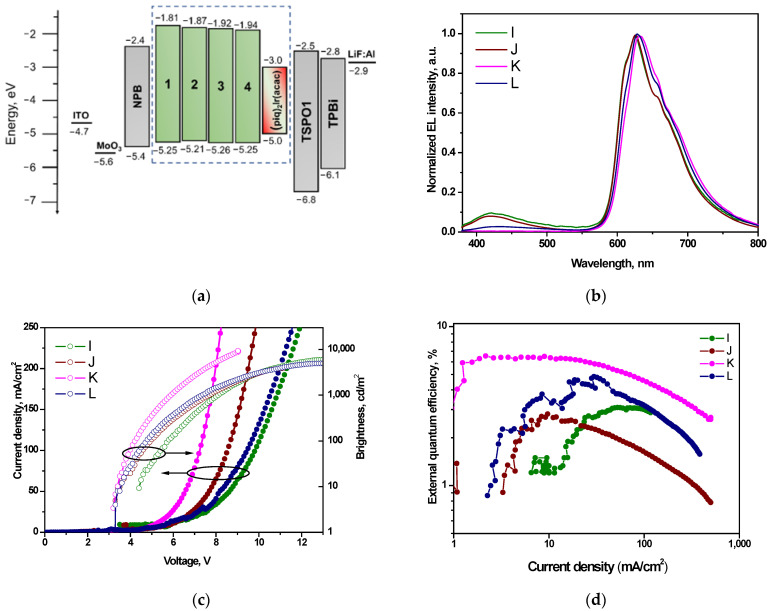
Diagram of energy levels of OLEDs I–L (**a**); electroluminescence spectra of OLEDs I–L recorded at applied voltage of 10 V (**b**); current density and luminance versus voltage plots (**c**); external quantum efficiency versus brightness plots (**d**). Arrow denotes the correspondence of data to axis.

To study the performance of compounds **1**–**4** as hosts, the simple PHOLEDs E-H and I-L were fabricated using green or red phosphorescent emitters, i.e., tris [2-phenylpyridinato-C2,N]iridium(III) (Ir(ppy)_3_) or bis [2-(1-isoquinolinyl-N)phenyl-C](2,4-pentanedionato-O2,O4)iridium(III) ((piq)_2_Ir(acac)), respectively. The concentration of emitters in the hosts was of 10 wt.%. The device structures and energy levels of the materials used in the devices are schematically shown in Figure 6a and Figure S14. The electroluminescence and efficiency characteristics of the devices E–H are given in Table 3 and Figure 6 and Figure S15.

Despite the exploitation of the green phosphorescent emitter Ir(ppy)_3_, most of the PHOLEDs of the series E–H were not characterized by green emission (Figure S16). Low-intensity violet emission bands were observed in EL spectra of PHOLEDs E–H. The wavelengths of these emission bands were close to those of fluorescence bands of pure **1**–**4**. Thus, the recombination of excitons occurred not only in the emitter but also in hosts. This observation can be attributed to inefficient host–guest energy transfer. Device G containing compound **3** as a host was characterized by green emission with the intensity maximum at ca. 510 nm, confirming the radiative recombination of excitons mainly on Ir(ppy)_3_. Thus, device G was characterized by the most efficient host–guest energy transfer. PHOLEDs E–H were characterized by the relatively low values of V_on_ (3.1–5.2 V), confirming the very efficient injection from the electrodes and transport of holes and electrons to the emitting layer. The highest brightness of 24,600 cd/m^2^ and the lowest V_on_ of 3.1 V observed for device G compared to those recorded for the other devices shows more effective exciton recombination and radiative transition in the emitting layer with host **3**. Thus, compound **3** can be regarded as the effective host for PHOLEDs. Device G with host **3** exhibited the maximum current, power, and external quantum efficiencies of 31 cd/A, 13.7 lm/W, 8.3%, respectively, in the absence of light outcoupling enhancement. It has to be noted that the simple and unoptimized PHOLED based on host **3** was fabricated. Therefore, the maximum efficiency of device was lower compared to those reported for other PHOLEDs containing Ir(ppy)_3_ [57].

Similarly, the highest maximum EQE of 6.4% was obtained for the red PhOLED (device K) based on host **3**. In contrast to other red PhOLEDs fabricated in this work, emission from the host was not observed in EL spectra of device K recorded at the different voltages. This observation demonstrates perfect host–guest energy transfer from **3** to (piq)_2_Ir(acac) (Figure 6a and Figure S17). Red PHOLEDs I–K also showed low turn-on voltages of 3.2–4.4 V (Figure 6c, Table 3). Maximum brightness of 8400 cd/m^2^ was observed for device K. This device exhibited maximum quantum efficiency of 6.4 % (Figure 6d).

**Figure 7:**
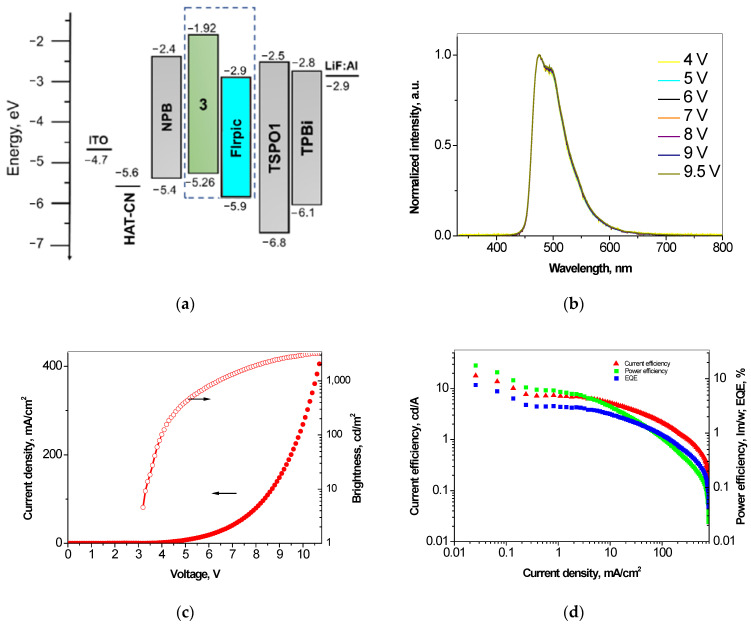
Diagram of energy levels (**a**); electroluminescence spectra of OLEDs recorded at different applied voltage (**b**); current density and luminance versus voltage plots (**c**); current efficiency, power efficiency, external quantum efficiency versus brightness plots (**d**) of PHOLED M. Arrow denotes the correspondence of data to axis.

Taking into account that **3** showed the best performance among the compounds of the series, PHOLED M was fabricated with the same structure as that of the red PHOLEDs except the layer of hexaazatriphenylenehexacarbonitrile (HAT-CN) and the layer of FIrpic doped in **3** as an emitting layer (Figure 7). The V_on_ of 3.2 V (Figure 7c) and appropriate spectral properties show complete electronic excitation energy transfer from **3** to FIrpic. Device M with a sky-blue EL reached of maximum EQE of 7.6% (Figure 7d).

## 4. Conclusions

Four new derivatives of carbazole and diphenyl imidazole were synthesized and characterized. Optical, photophysical, electrochemical, and photoelectric properties of the compounds were found to be similar, indicating that attachment of the additional carbazole–diphenyl imidazole fragment has no significant impact on these properties. Compounds with two imidazole rings demonstrated higher thermal stability and glass transition temperatures compared with those of the derivatives having one imidazole ring. OLEDs fabricated using the synthesized compounds as emitters showed deep-blue and blue emissions with maximum quantum efficiency ranging from 0.6% to 1.1%. The suitable values of energy levels, triplet energies and charge carrier mobilities allowed the use of the synthesized compounds as hosts for green and red phosphorescent OLEDs. The devices showed sky-blue, green or red emissions, confirming the radiative recombination of excitons in phosphorescent emitters. Maximum quantum efficiencies of 7.6%, 8.3%, and 6.4%, respectively, were observed without optimization of the structures.

## Figures and Tables

**Table 1:** Thermal and photophysical characteristics of compounds **1**–**4**.

Compound	*T_D_*, °C	*T_m_*, °C	*T_g_*, °C	*T_cr_*, °C	*λ_ab_*, nm	*λ_fl_*, nm	*E_T_*, eV
**1**	399	270	134	236	299	398	3.18
**2**	400	-	147	-	303	398	3.16
**3**	477	-	151	-	303	402	3.08
**4**	487	-	172	-	304	403	3.05

*T_D_*—5% weight-loss temperature; *T_m_*—melting point; *T_g_*—glass transition temperature; *T_cr_*—crystallization temperature, *λ_ab_*—lowest energy band of absorption; *λ_fl_*—wavelength of maximum intensity of fluorescence of the solutions in THF; *E_T_*—triplet energy determined from phosphorescence spectrum.

**Table 2:** Electrochemical and photoelectrical characteristics of compounds **1**–**4**.

Compound	IP_CV_ ^1^, eV	EA_CV_, eV	IP_PE_ ^2^, eV	μ_h_, cm^2^/V·s ^3^	μ_e_, cm^2^/V·s ^3^
**1**	5.25	1.81	5.35	2.9 × 10^−4^	-
**2**	5.21	1.87	5.18	3.8 × 10^−5^	-
**3**	5.26	1.92	5.29	1.1 × 10^−4^	-
**4**	5.25	1.94	5.22	6.3 × 10^−5^	1.2 × 10^−4^

^1^ Determined by cyclic voltammetry; ^2^ measured by UV photoelectron spectrometry in air; ^3^ charge mobility at 6.4 × 10^5^ V/cm.

**Table 3:** Electroluminescent characteristics of devices A–M.

OLED	Emitting Layer (EML)	λ_em_, nm	CIE, xy	V_on_, V	Max. Brightness, cd/m^2^	Max. Current Efficiency, cd/A	Max. Power Efficiency, lm/W	Max. EQE, %
Doping-free blue fluorescent devices ITO/MoO_3_/NPB/EML/TSPO1/TPBi/LiF/Al								
A	**1**	420	(0.17, 0.09)	4.0	840	0.7	0.38	0.9
B	**2**	418	(0.16, 0.08)	4.1	1030	0.8	0.42	1.1
C	**3**	431	(0.17, 0.15)	4.8	1350	0.9	0.43	0.8
D	**4**	437	(0.17, 0.14)	4.0	1430	0.8	0.41	0.6
Green phosphorescent devices ITO/m-MTDATA/EML/Bphen/LiF/Al								
E	Ir(ppy)_3_:**1**	422	(0.21, 0.17)	5.2	5580	2.7	1.1	2.5
F	Ir(ppy)_3_:**2**	510	(0.27, 0.58)	3.7	4630	3.5	1.6	0.97
G	Ir(ppy)_3_:**3**	510	(0.29, 0.60)	3.1	24,600	31.1	13.7	8.3
H	Ir(ppy)_3_:**4**	510	(0.25, 0.39)	3.9	5800	1.9	1.1	0.74
Red phosphorescent devices ITO/MoO_3_/NPB/EML/TSPO1/TPBi/LiF/Al								
I	(piq)_2_Ir(acac):**1**	625	(0.56, 0.29)	4.4	5700	2.7	0.9	3.0
J	(piq)_2_Ir(acac):**2**	625	(0.59, 0.29)	4.0	4800	2.6	1.4	2.7
K	(piq)_2_Ir(acac):**3**	632	(0.67, 0.32)	3.2	8400	4.4	3.3	6.4
L	(piq)_2_Ir(acac):**4**	629	(0.63, 0.31)	3.3	4700	3.9	1.7	4.5
Sky-blue phosphorescent device ITO/HAT-CN/NPB/EML/TSPO1/TPBi/LiF/Al								
M	FIrpic:**3**	475	(0.15, 0.36)	3.2	3300	17.9	17.6	7.6

## Data Availability

All experimental data to support the findings of this study are available upon request by contacting the corresponding authors.

## Supplementary Materials

The following supporting information can be downloaded at: https://www.mdpi.com/article/10.3390/ma15238495/s1. Figure S1. (a) TGA curves of compounds **2** and **4**; (b–d) DSC curves of compounds **2**–**4**. Figure S2. Absorption (a) and PL (b) spectra of **1** solutions. Figure S3. Absorption (a) and PL (b) spectra of **2** solutions. Figure S4. Absorption (a) and PL (b) spectra of **3** solutions. Figure S5. Absorption (a) and PL (b) spectra of **4** solutions. Figure S6. Lippert–Mataga plots for **1** (a), **3** (b) and **4** (c). Figure S7. (a–c) Phosphorescence spectrum of dilute solutions of **1**–**3** in THF at 77 K. Figure S8. PL decay curves of solutions of **1**–**4** in THF. Figure S9. PL spectra of THF solutions of **1** (a) and **3** (b), normalized PL spectra of **3** (c) before and after 40 min photoexcitation by the UV light. Figure S10. (a–d) Cyclic voltammograms of compounds **1**–**4**. Figure S11. TOF signals for holes/electrons in vacuum-deposited layers of compounds **1**–**4** at different electric fields. Figure S12. EL spectra of non-doped devices A–D at different voltages. Figure S13. Electroluminescence characteristics of OLEDs A–D: (a) current efficiency versus current density, (b) power efficiency versus current density. Figure S14. Diagram of energy levels of green PHOLEDs. 4,4′,4′′-tris[phenyl(m-tolyl)amino]triphenylamine (m-MTDATA) and bathophenanthroline (Bphen) were used as hole and electron transporting layers, respectively. Figure S15. Electroluminescence characteristics of PHOLEDs E–H: (a) current density and luminance versus voltage, (b) current efficiency versus current density, (c) power efficiency versus current density, (d) external quantum efficiency versus current density. Figure S16. Electroluminescence spectra of PHOLEDs E–H recorded at applied voltage of 10 V. Figure S17. EL spectra of devices I–L at different voltages. Refs. [58,59] are cited in supplementary materials.
