# Supplementary material for: Derivatives of Imidazole and Carbazole as Bifunctional Materials for Organic Light-Emitting Diodes
Source: Materials (Basel). 2022 Nov 29;15(23):8495. doi: 10.3390/ma15238495 (PMC9739035; doi:10.3390/ma15238495)
Supplement: Supplementary file 1 [file materials-15-08495-s001.zip › materials-2059656-supplementary.pdf]

DERIVATIVES OF IMIDAZOLE AND CARBAZOLE AS BIFUNCTIONAL MATERIALS FOR ORGANIC LIGHT-EMITTING DIODES

Oleksandr Bezvikonnyi, Ronit Sebastine Bernard, Viktorija Andruleviciene, Dmytro Volyniuk, Rasa Keruckiene, Kamile Vaiciulaityte, Juozas Vidas Grazulevicius *

Department of Polymer Chemistry and Technology, Kaunas University of Technology, K. Baršausko g. 59, LT- 51423, Kaunas, Lithuania

1.1. Instrumentation

^{1}H NMR and ^{13}C NMR spectra were obtained of solutions in deuterated chloroform ($CDCl_3$-*d*$_6$) or dimethyl sulfoxide (DMSO-*d*$_6$,) with a Bruker Avance III spectrometer. The data are given as chemical shifts in δ (ppm) and tetramethylsilane was used as an internal standard. IR spectra were recorded by a Vertex 70 Bruker spectrometer equipped with an ATR attachment with a diamond crystal over frequencies of 600–3500 cm^{-1} with a resolution of 5 cm^{-1} over 32 scans. Mass spectra were obtained on a Waters ZQ 2000 mass spectrometer. Melting point (mp) was determined using Electrothermal Mel-Temp melting point apparatus. Thermogravimetric analysis (TGA) was performed on a TA Instruments Q50 apparatus at a heating rate of 20 ºC/min under nitrogen atmosphere. Differential scanning calorimetry (DSC) measurements were performed on a 100 TA Instruments Q2000 thermal analyzer at a heating/cooling rate of 10 ºC/min under nitrogen atmosphere. Photophysical properties of 10^{-4} M solutions in tetrahydrofuran and pure layers of the synthesized compounds were recorded using a Perkin Elmer Lambda 35 and an Edinburgh Instruments FLS980 spectrometers. Cyclic voltammetry (CV) measurements were carried out using an Autolab potentiostat PGSTAT20 in a three electrode cell using platinum rod as a counter electrode, glassy carbon as working

electrode and silver wire as the reference electrode. The experiments were carried out in dry dichloromethane solution containing 0.1 M tetrabutylammonium perchlorate as electrolyte at room temperature under nitrogen atmosphere at a scan rate 50 mV/s. The measurements were calibrated using the internal standard ferrocene/ferrocenium (Fc/Fc^+). The ionization potentials (IP_{PE}) of the films of the synthesized compounds were measured by UV photoelectron spectrometry in air[1]. Hole drift mobility of the materials was estimated by a time-of-flight (TOF) method. The samples were fabricated with structures of ITO/compound/Al. The thicknesses of the layers for TOF measurements were 2.6, 3.2, 2.7, 5.4 μm for 1, 2, 3 and 4, respectively. Commercial indium tin oxide (ITO)-coated glass was used as a substrate, which was first cleaned chemically using a bath of distilled water and acetone. Organic and Al films were sequentially vacuum-deposited onto precleaned ITO-coated glass substrates under the vacuum of $2\text{-}5\cdot10^{-6}$ mBar using vacuum equipment from Kurt J. Lesker in-built in an MB EcoVap4G glove box. In the time of flight (TOF) experiments, charges were generated by a pulsed third-harmonic Nd:YAG laser EKSPLA NL300 working at a pulse duration of 3–6 ns and the wavelength of 355 nm. Electric fields were applied by a Keithley 6517B electrometer. A digital storage oscilloscope Tektronix TDS 3032C was used to record TOF transients. The drift mobility was calculated by using the formula $\mu=d^2/U\cdot t_t$, where d is the layer thickness, U the surface potential at the moment of illumination, and t_t is the transit time which was taken from the TOF transients. Current–voltage curves were recorded using a Keithley 2400 Source Meter. A xenon lamp fitted with an AM1.5 global filter was used for illumination. Silicon photodiode was used to calibrate the light source to give 1 sun light intensity. Voltage was cyclically changed from 0 to 1 V and to -1 V under illumination or in the dark. A Keithley source meter 2400-C was utilized for recoding of the current density–voltage characteristics. The current density–luminance characteristics were estimated using a calibrated silicon photodiode with the 6517B Keithley electrometer. Electroluminescence (EL) spectra were

recorded by an Avantes AvaSpec-2048XL spectrometer. The current, power and external quantum efficiencies were estimated utilizing the current density, luminance, and EL spectra as reported earlier [2].

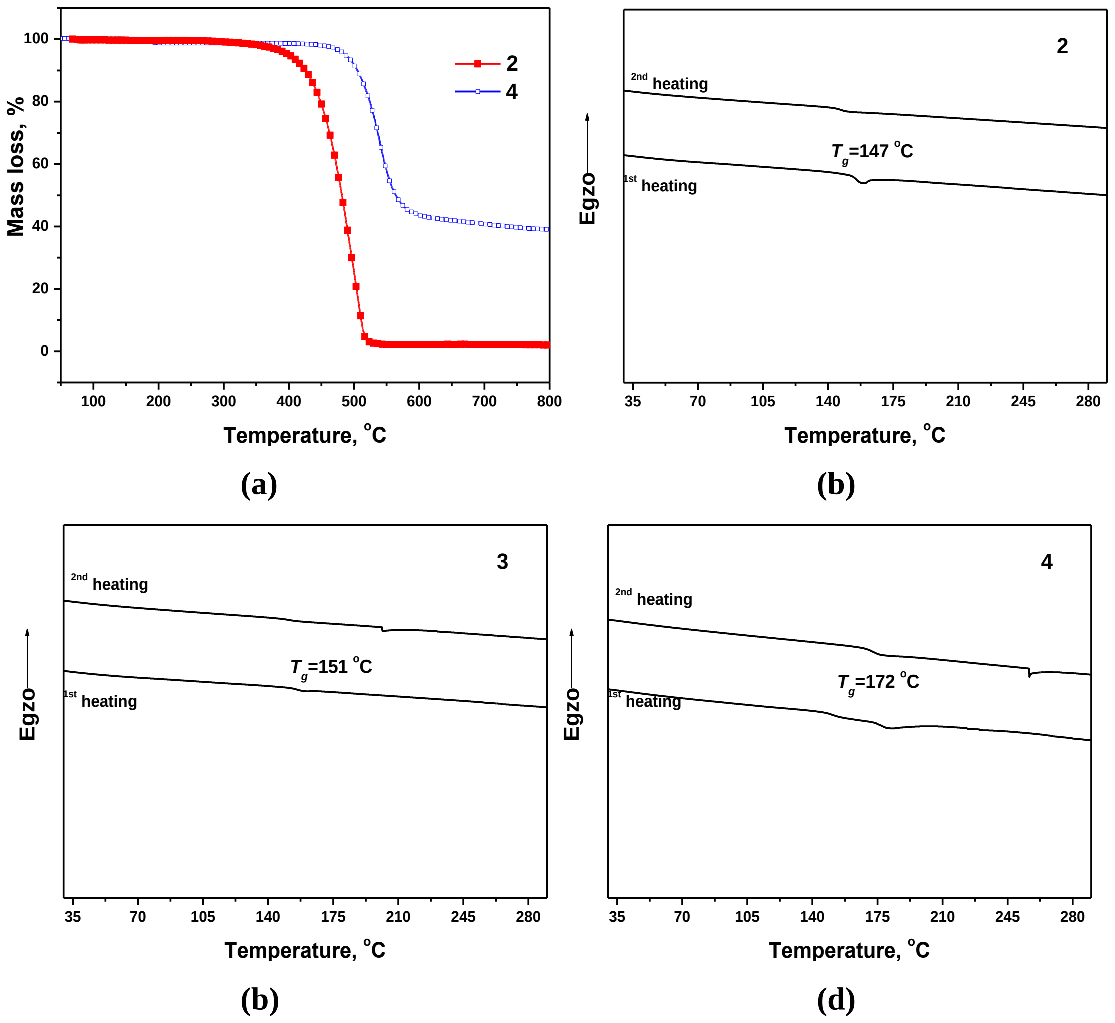

Figure S1. (a) TGA curves of compounds **2** and **4**; **(b-d)** DSC curves of compounds **2–4**

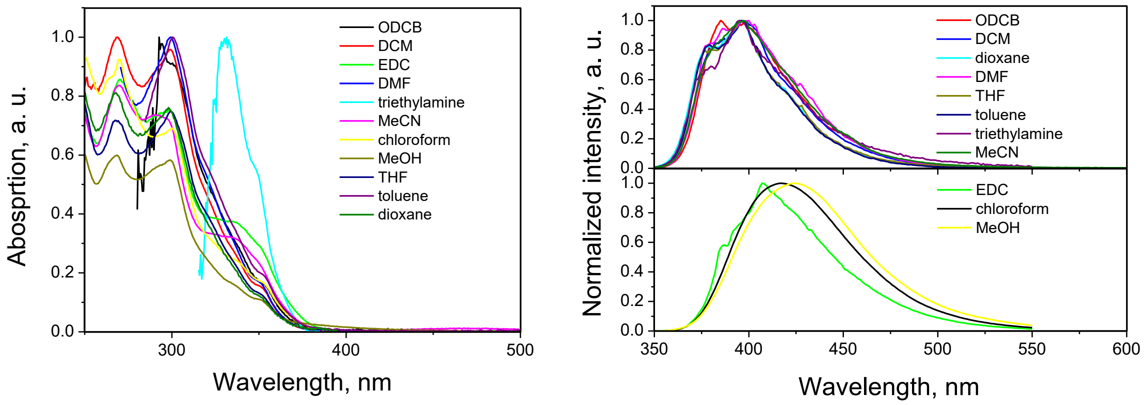

(a) **(b)**

Figure S2. Absorption **(a)** and PL **(b)** spectra of **1** solutions

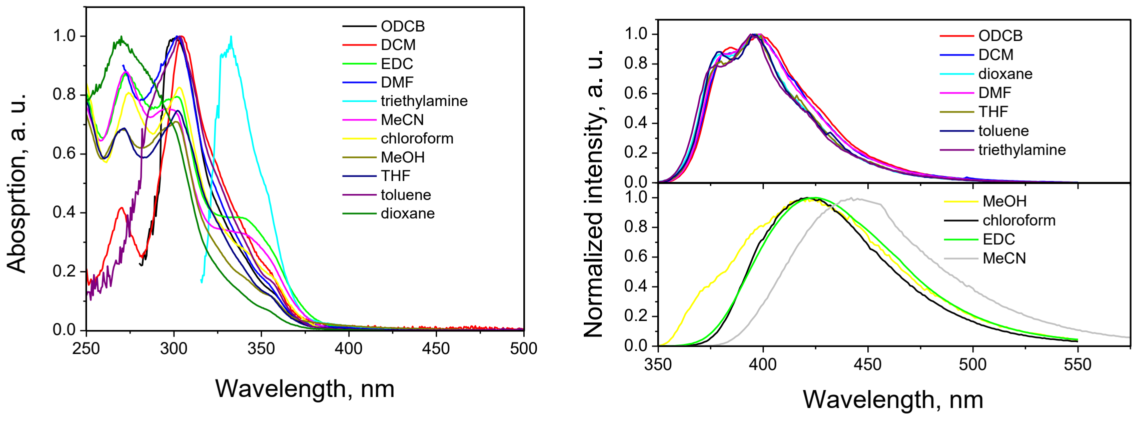

(a) **(b)**

Figure S3. Absorption **(a)** and PL **(b)** spectra of **2** solutions

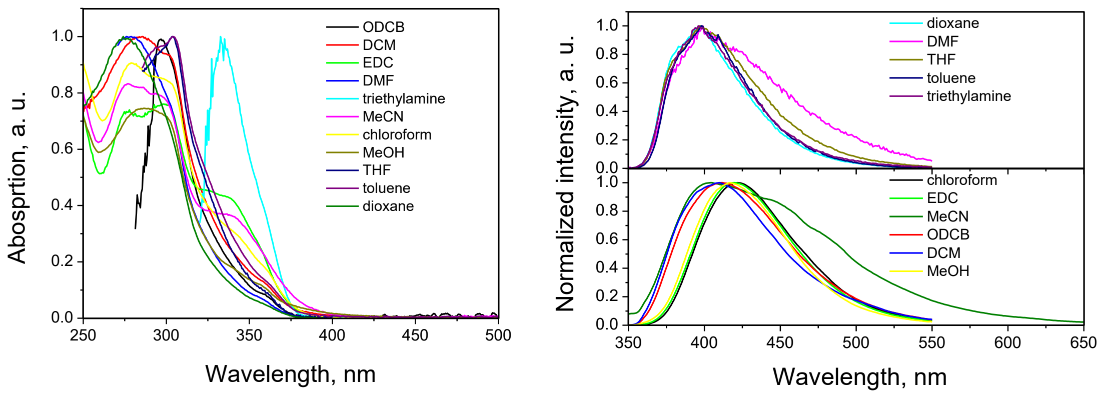

(a) **(b)**

Figure S4. Absorption **(a)** and PL **(b)** spectra of **3** solutions

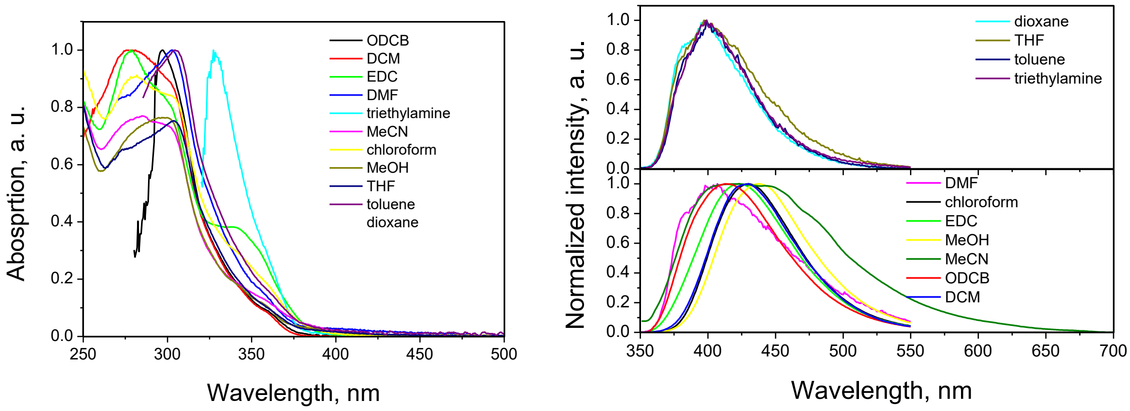

(a) **(b)**

Figure S5. Absorption **(a)** and PL **(b)** spectra of **4** solutions

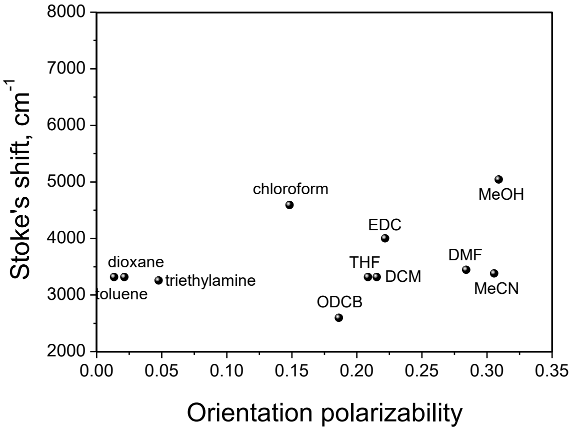

(a)

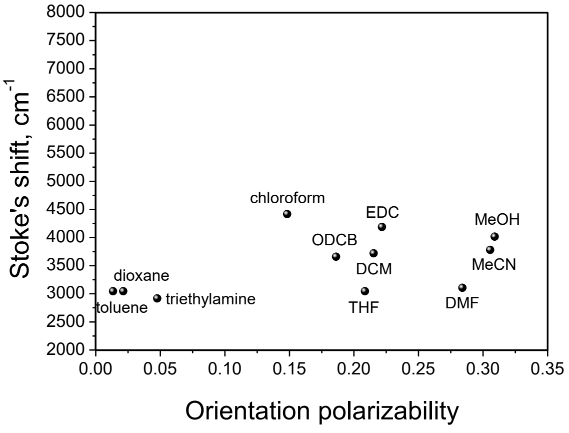

(b)

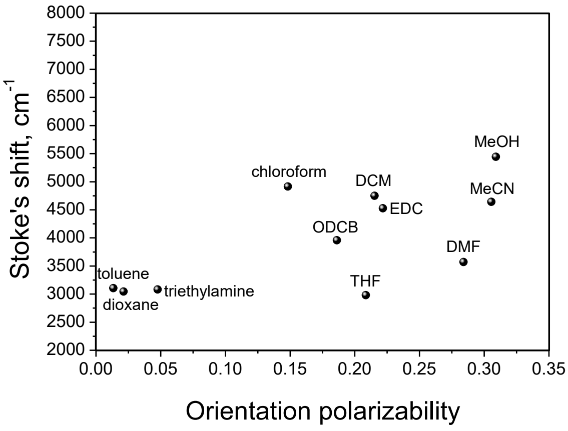

(c)

Figure S6. Lippert–Mataga plots for **1** (**a**), **3** (**b**) and **4** (**c**)

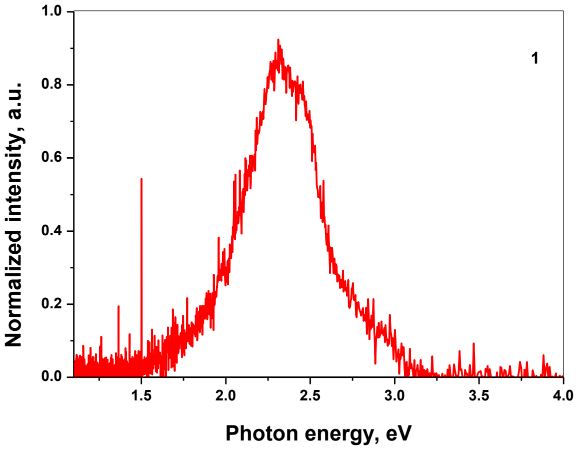

(b)

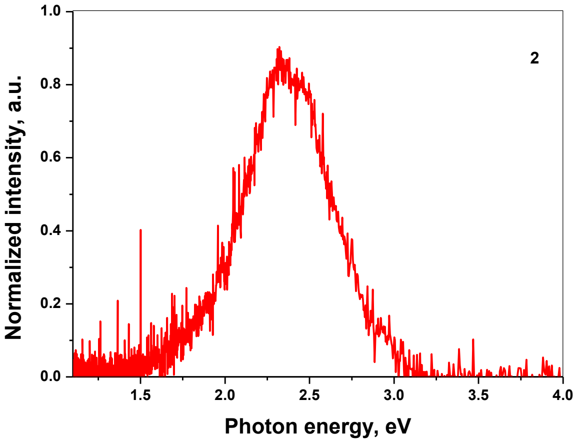

(b)

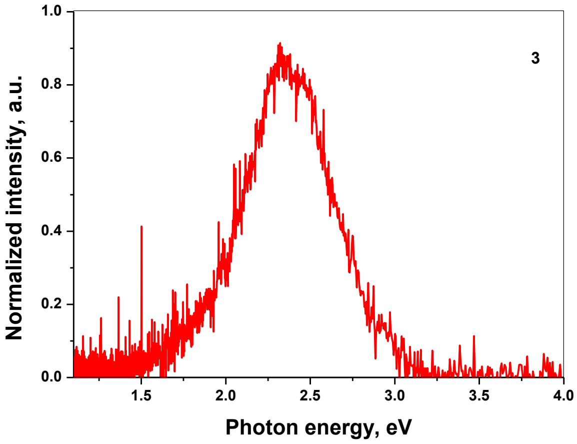

(c)

Figure S7. (a-c) Phosphorescence spectrum of dilute solutions of **1**–**3** in THF at 77K

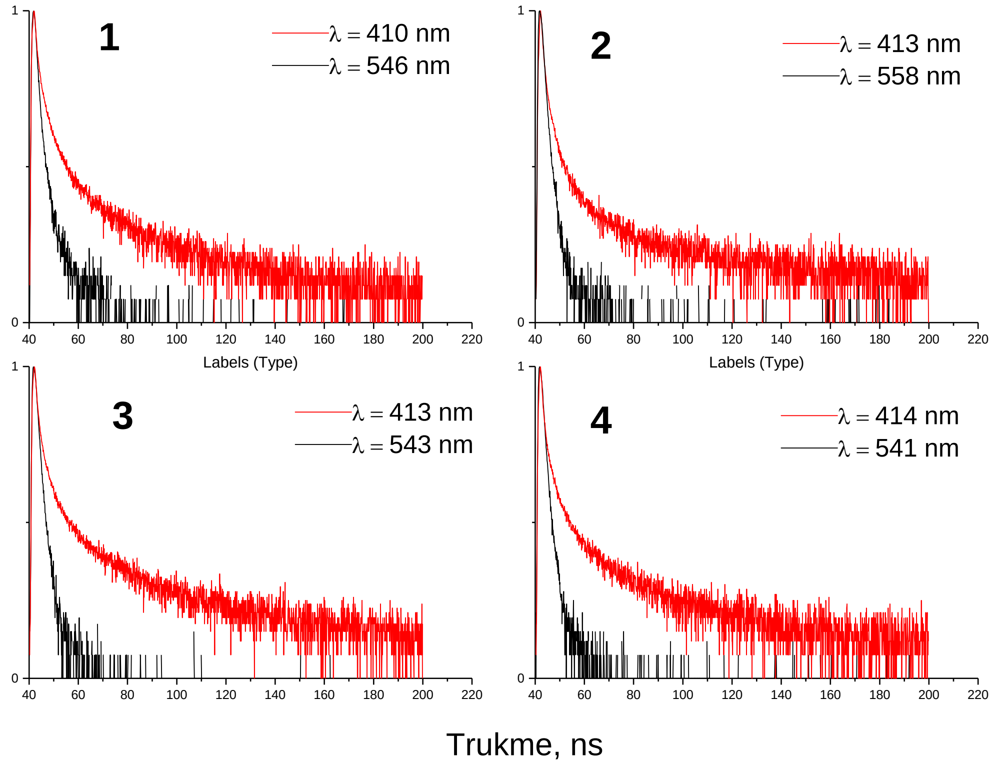

Figure S8. PL decay curves of solutions of **1**–**4** in THF

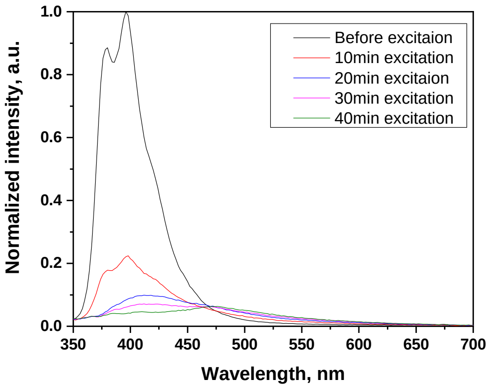

(a)

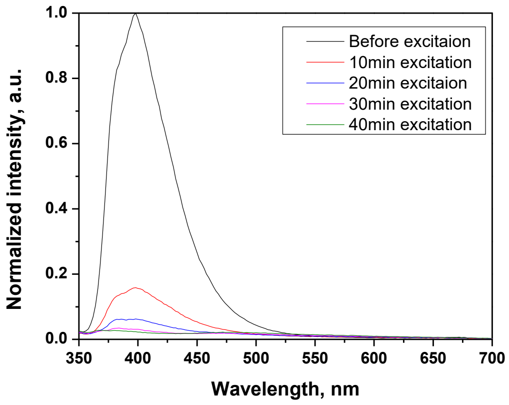

(b)

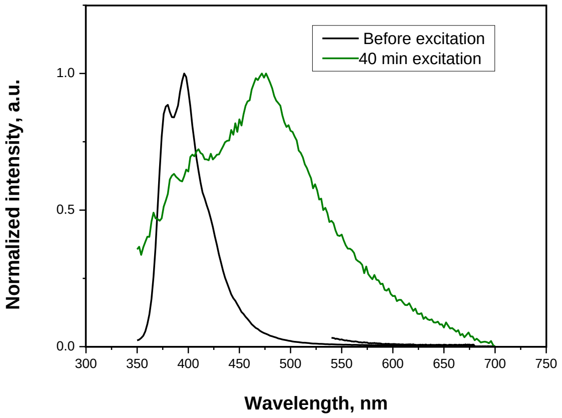

(c)

Figure S9. PL spectra of THF solutions of **1** (a) and **3** (b), normalized PL spectra of **3** (c) before and after 40 min photoexcitation by the UV light

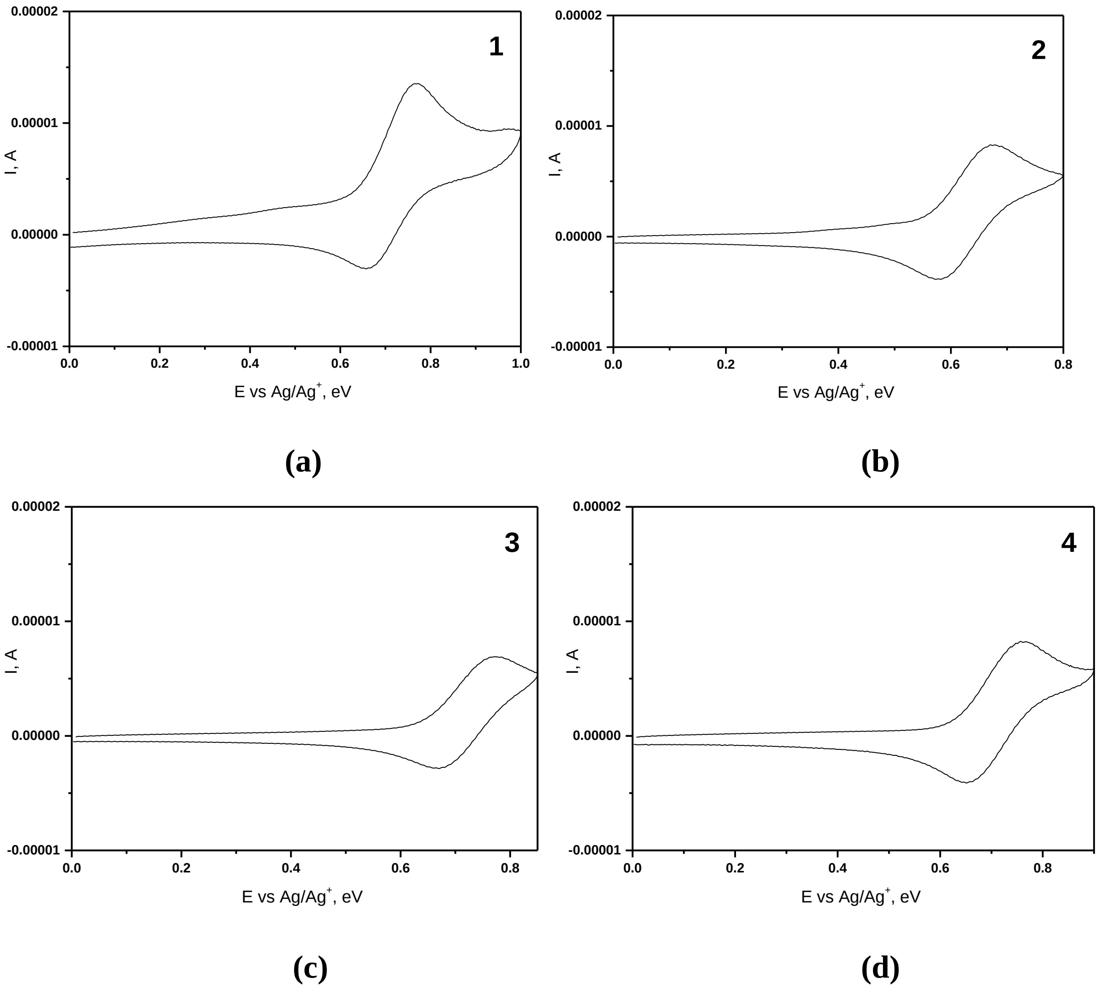

Figure 10. (a-d) Cyclic voltammograms of compounds **1–4**

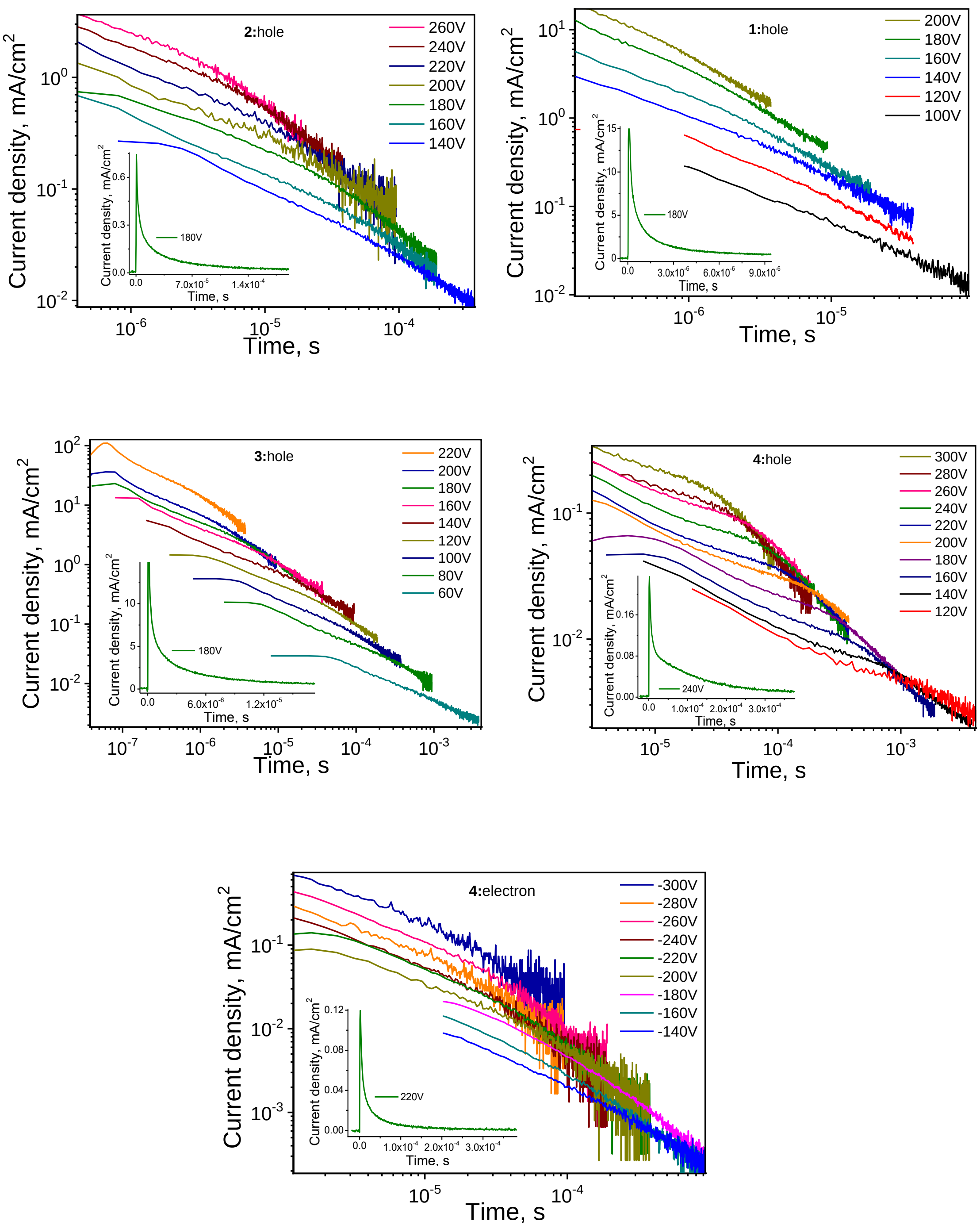

Figure S11. TOF signals for holes/electrons in vacuum-deposited layers of compounds **1**–**4** at different electric fields**.**

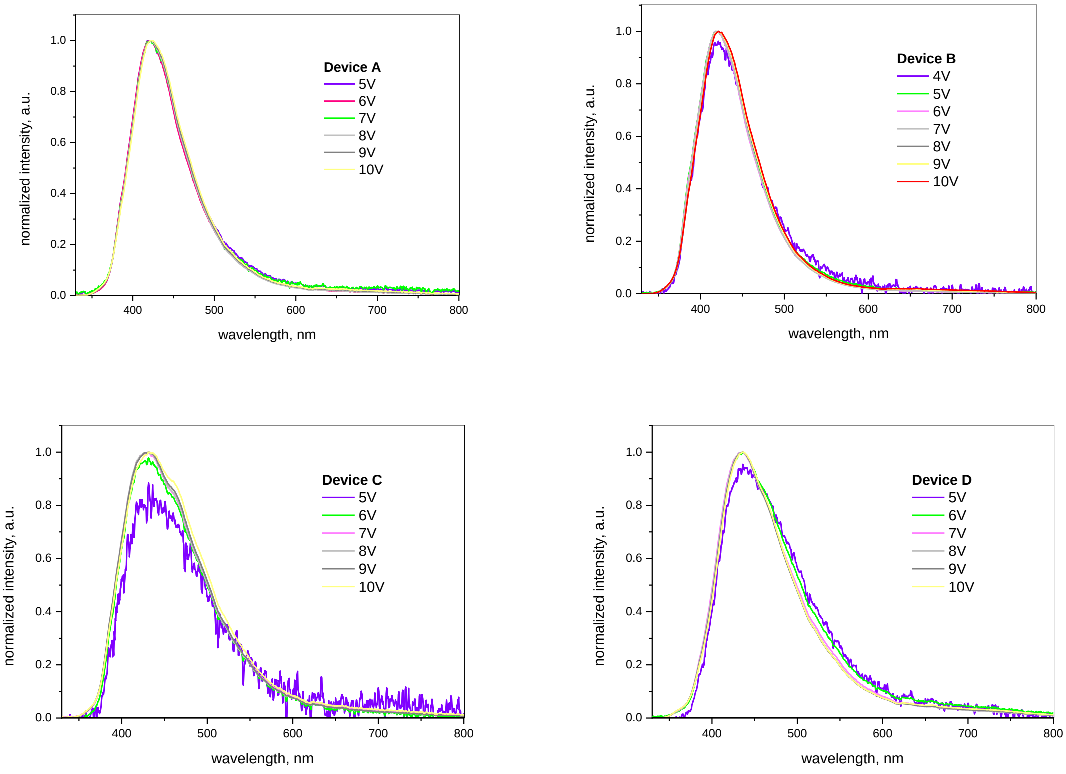

Figure S12. EL spectra of non-doped devices A–D at different voltages.

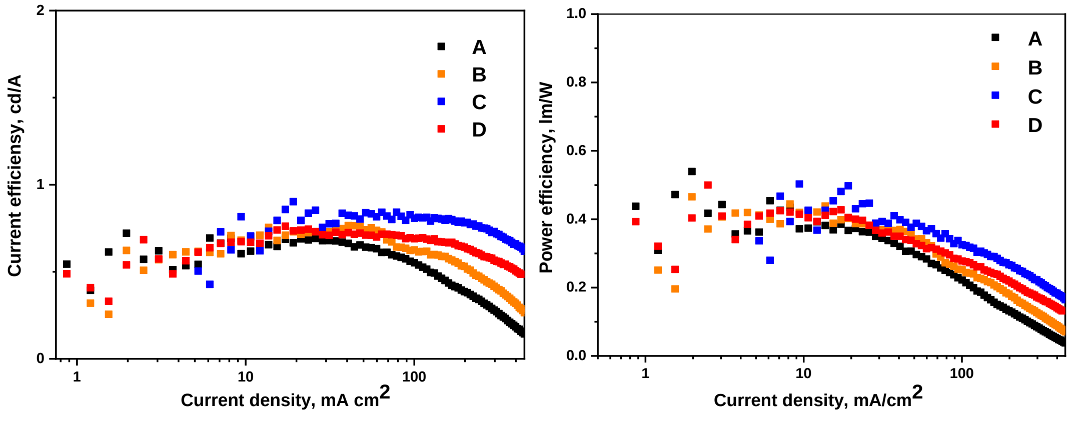

(a) **(b)**

Figure S13. Electroluminescence characteristics of OLEDs **A-D**: **(a)** current efficiency versus current density, **(b)** power efficiency versus current density.

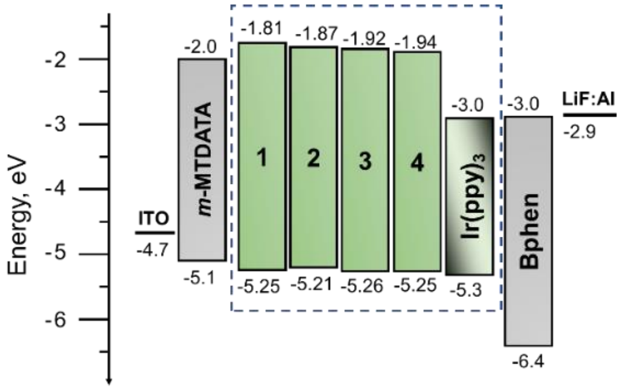

Figure S14. Diagram of energy levels of green PHOLEDs. 4,4′,4″-tris[phenyl(m-tolyl)amino]triphenylamine (m-MTDATA) and bathophenanthroline (Bphen) were used as hole and electron transporting layers, respectively.

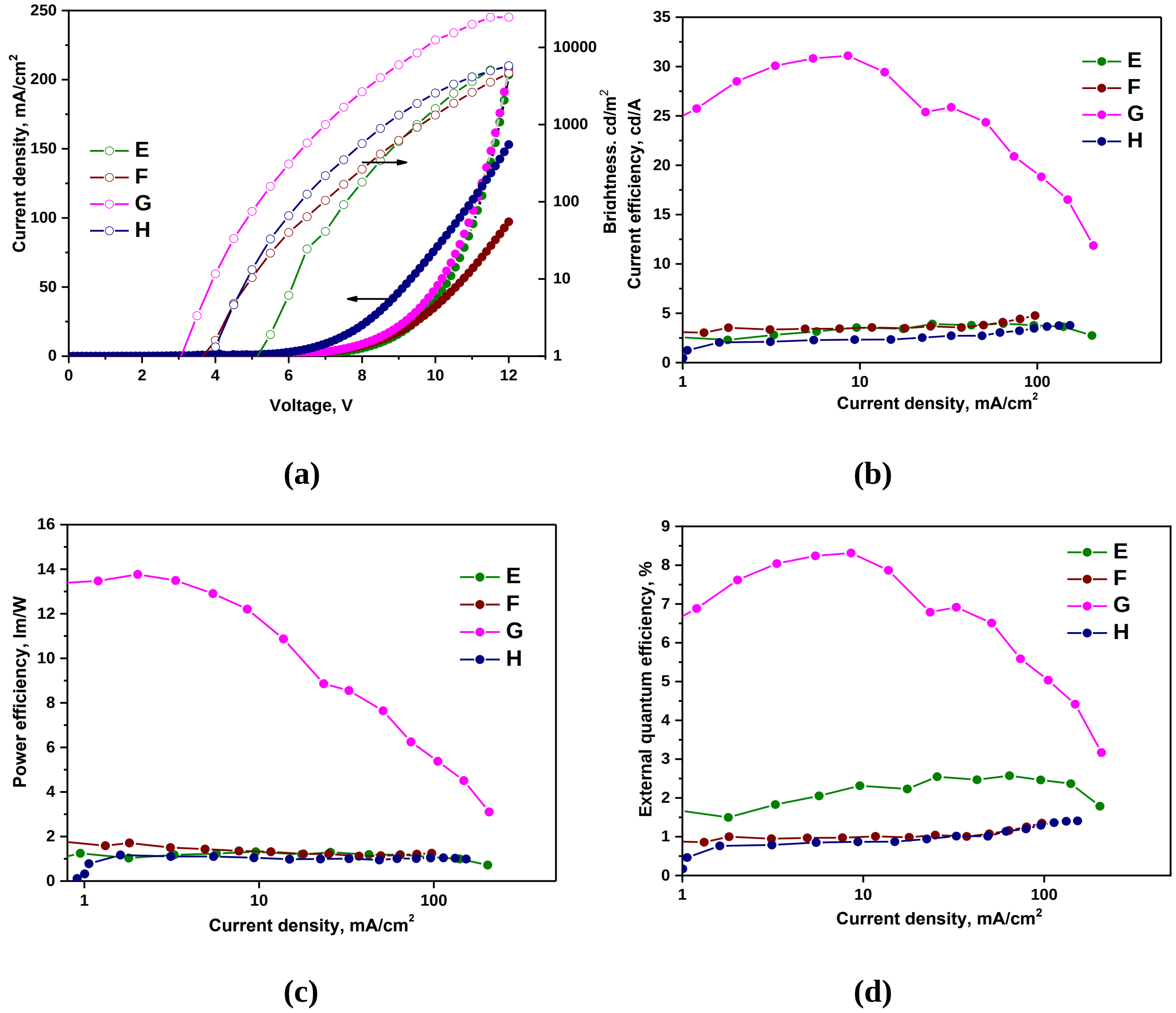

Figure S15. Electroluminescence characteristics of PHOLEDs **E**–**H**: **(a)** current density and luminance versus voltage, **(b)** current efficiency versus current density, **(c)** power efficiency versus current density, (**d**) external quantum efficiency versus current density

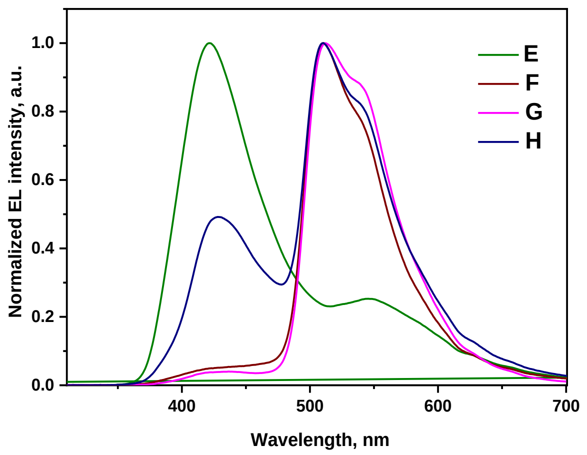

Figure S16. Electroluminescence spectra of PHOLEDs **E**–**H** recorded at applied voltage of 10 V.

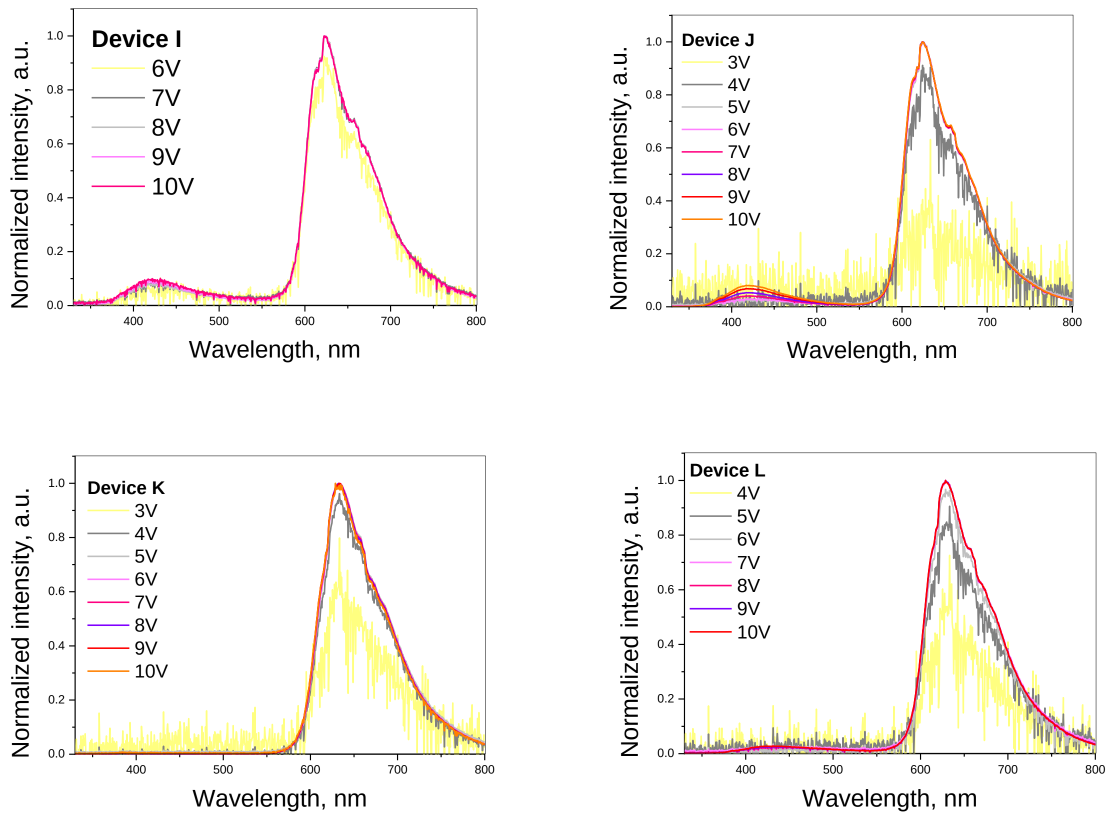

Figure S17. EL spectra of devices I–L at different voltages.

References

[1] Miyamoto, E.; Yamaguchi, Y.; Yokoyama, M. Ionization Potential of Organic Pigment Film by Atmospheric Photoelectron Emission Analysis. *Electrophotography* **1989**, *28*, 364–370. https://doi.org/10.11370/isjepj.28.364.

[2] Okamoto, S.; Tanaka, K.; Izumi, Y.; Adachi, H.; Yamaji, T.; Suzuki, T. Simple measurement of quantum efficiency in organic electroluminescent devices. *Jpn. J. Appl. Phy. Part 2 Lett.* **2001**, *40*, 783–784. https://doi.org/10.1143/jjap.40.l783.
